# Predictive value of magnetic resonance imaging-based texture analysis for hemorrhage transformation in large cerebral infarction

**DOI:** 10.3389/fnins.2022.923708

**Published:** 2022-07-22

**Authors:** Heng Zhai, Zhijun Liu, Sheng Wu, Ziqin Cao, Yan Xu, Yinzhang Lv

**Affiliations:** ^1^Department of Neurology, Union Hospital, Tongji Medical College, Huazhong University of Science and Technology, Wuhan, China; ^2^Department of Radiology, Union Hospital, Tongji Medical College, Huazhong University of Science and Technology, Wuhan, China; ^3^Department of Chemistry, Emory University, Atlanta, GA, United States; ^4^Department of Radiology, Tongji Hospital, Tongji Medical College, Huazhong University of Science and Technology, Wuhan, China

**Keywords:** massive cerebral infarction, hemorrhagic transformation, prediction, texture analysis, DWI, T2/FLAIR

## Abstract

Massive cerebral infarction (MCI) is a devastating condition and associated with high rate of morbidity and mortality. Hemorrhagic transformation (HT) is a common complication after acute MCI, and often results in poor outcomes. Although several predictors of HT have been identified in acute ischemic stroke (AIS), the association between the predictors and HT remains controversial. Therefore, we aim to explore the value of texture analysis on magnetic resonance image (MRI) for predicting HT after acute MCI. This retrospective study included a total of 98 consecutive patients who were admitted for acute MCI between January 2019 and October 2020. Patients were divided into the HT group (*n* = 44) and non-HT group (*n* = 54) according to the follow-up computed tomography (CT) images. A total of 11 quantitative texture features derived from images of diffusion-weighted image (DWI) or T2-weighted-Fluid-Attenuated Inversion Recovery (T2/FLAIR) were extracted for each patient. Receiver operating characteristic (ROC) analysis were performed to determine the predictive performance of textural features, with HT as the outcome measurement. There was no significant difference in the baseline demographic and clinical characteristics between the two groups. The distribution of atrial fibrillation and National Institutes of Health Stroke Scale (NIHSS) were significantly higher in patients with HT than those without HT. Among the textural parameters extracted from DWI images, six parameters, f2 (contrast), f3 (correlation), f4 (sum of squares), f5 (inverse difference moment), f10 (difference variance), and f11 (difference entropy), differs significantly between the two groups (*p* < 0.05). Moreover, five of six parameters (f2, f3, f5, f10, and f11) have good predictive performances of HT with the area under the ROC curve (AUC) values of 0.795, 0.779, 0.791, 0.780, and 0.797, respectively. However, the texture features f2, f3, and f10 in T2/FLAIR images were the only three significant predictors of HT in patients with acute MCI, but with a relatively low AUC values of 0.652, 0.652, and 0.670, respectively. In summary, our preliminary results showed DWI-based texture analysis has a good predictive validity for HT in patients with acute MCI. Multiparametric MRI texture analysis model should be developed to improve the prediction performance of HT following acute MCI.

## Introduction

Massive cerebral infarction (MCI), which accounts for 10–15% of all acute ischemic stroke (AIS) cases worldwide (Huttner and Schwab, 2009), is a devastating condition caused by complete occlusion of the internal carotid artery trank or middle cerebral artery trank, or their cortical branches. The overall prognosis for MCI is poor, with the mortality rate of patients with conservative medical treatments as high as 53–78% and most survivors left with severe disabilities (Su et al., 2016; Lin and Frontera, 2021). There is no unified diagnostic criteria for MCI and the Adams’ classification method is often used, i.e., the focal site affects more than two anatomical parts, and the diameter of infarct lesions is above or equal to 3 cm (Adams et al., 1993).

It is well-known that stroke-related complications during hospitalization are the leading cause of death, accounting for approximate 20–50% of all deaths in ischemic stroke patients (Ekeh et al., 2015). Stroke-related complications include common in-hospital medical complications as well as neurological complications, such as cerebral edema, intracranial hypertension, hemorrhagic transformation (HT), recurrent stroke, and poststroke seizures (Li et al., 2019). HT is as a frequent and serious complication in patients with AIS, especially those with acute MCI, and severe HT is associated with deteriorating neurological symptoms and poor outcomes (Su et al., 2016). HT can be subdivided into two major subtypes, hemorrhagic infarction and parenchymal hematoma, with respect to the type of hemorrhage. Although, the pathophysiological mechanism of HT is still unclear, hypotheses involving the loss of microvascular integrity and disruption of neurovascular homeostasis have been proposed (Wang and Lo, 2003; Arba et al., 2020).

Previous studies have reported several risk factors or predictors for HT after AIS, including the use of antiplatelets or anticoagulants, size of infarction, demographic factors, atrial fibrillation, hypertension, diabetes mellitus, lipid profile, National Institutes of Health Stroke Scale (NIHSS), reperfusion therapy, and white matter hyperintensity burden (Paciaroni et al., 2008; Thomas et al., 2021). Good performance of Magnetic Resonance Imaging (MRI) techniques, such as T2-weighted-Fluid-Attenuated Inversion Recovery (T2/FLAIR), diffusion-weighted imaging (DWI) and perfusion-weighted imaging (PWI), has also been reported for the prediction of HT in patients with AIS (Oppenheim et al., 2002; Jha et al., 2014; Shinoda et al., 2017). However, the diagnostic performance of MRI for predicting HT in acute MCI is uncertain. Understanding these risk factors and assessing predictors in depth can significantly help physicians develop strategies to reduce the occurrence of HT and also provide insights into the pathophysiological mechanism of disease.

A quantitative analysis, texture analysis, has been widely used for qualitative diagnosis, efficacy evaluation, and prognosis prediction in neurological disorders by virtue of its unique quantification characteristics of morphological features of tissues (Kassner and Thornhill, 2010). MRI-based texture analysis provides information related to the spatial distribution and intensity of gray levels over the regions of interest. Recently, texture analysis has been shown to predict HT in patients with AIS (Kassner et al., 2009; Yu et al., 2018).

To our knowledge, MRI-based texture analysis for prediction of HT in AIS patients, especially patients with MCI, has not yet been completely exploited. We hypothesized that texture analysis on MR images might be sensitive enough to predict HT in patients with acute MCI. Therefore, we applied gray-level co-occurrence matrix (GLCM) based texture analysis to conventional MR imaging of patients with acute MCI to determine whether MRI-based texture analysis can accurately predict the appearance of HT.

## Materials and methods

### Patients

**FIGURE 1:**
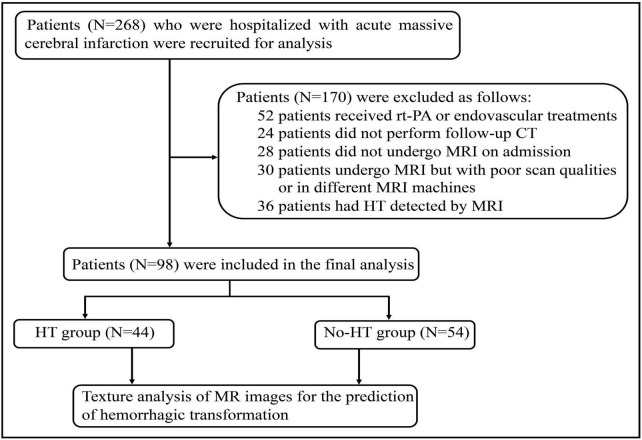
Flow diagram for patient selection.

This study was approved by the Ethics Committee of Union Hospital, Tongji Medical College of Huazhong University of Science and Technology. Informed consent was waived because of retrospective nature of this study. The study were also conducted in accordance with the Declaration of Helsinki. The clinical and radiological data were obtained retrospectively from relevant medical records between January 2019 and October 2020. The inclusion criteria were as follows: (1) patients without any type of intracranial hemorrhage or lesions that might mimic AIS on initial computed tomography (CT) at the time of symptom onset; (2) patients who did not receive recombinant tissue plasminogen activator (rt-PA) and endovascular treatment, (3) DWI performed at the time of hospitalization within 24 h; (4) HT detected by follow-up CT 72 h after initial imaging. Exclusion criteria included: (1) patients with evidence of intracerebral hemorrhage on initial CT; (2) patients who did not undergo MRI scan or follow-up CT after admission; (3) patients who was performed MRI scan using a different machine; (4) poor quality of MR images. Baseline demographic data including age, gender, and also smoking, current alcohol drinking, medical history of hypertension, diabetes mellitus, NIHSS score, laboratory findings, and treatment measures during hospitalization were collected as well. Two hundred and sixty eight acute MCI patients with complete data were initially analyzed and 98 patients, consisting of 44 patients who developed HT during hospitalization and 54 patients without HT, were included in the final analysis according to the criteria (Figure 1).

### Magnetic resonance imaging protocol and imaging analysis

All patients were performed on 1.5 Tesla MRI equipment (GE Healthcare, Milwaukee, WI, United States) using a 24-channel head coil. MRI techniques included T1WI, T2/FLAIR, and DWI. For the MRI protocol in this study were as follows: (1) T1WI sequence: matrix size = 256 × 192, field of view (FOV) = 240 × 240 mm, repetitive time (TR) = 1,800 ms, echo time (TE) = 20 ms, flip angle (FA) = 90, slice thickness = 4.3 mm. (2) T2-FLAIR sequence: TR/TE = 8,500/120 ms, matrix = 256 × 192, FA = 130°, slice thickness = 4.3 mm. (3) DWI scan uses spin-echo echo planar imaging (SE-EPI) sequence, b value is 0 and 1,000 s/mm^2^, TR = 7,600 ms, TE = 100 ms, FOV 240 × 240 cm, slice thickness = 4.3 mm, no slice gap and matrix size = 128 × 96. MR angiography (MRA) was used to assess the severity of intracranial arterial stenosis (ICAS). HT was categorized as hemorrhagic infarction (HI) or parenchymal hematoma (PH), both of these types can be classified into two subtypes. Hemorrhagic infarction was defined as small hemorrhagic petechiae located along the peripheral margins of the infarct (HI-1) or more confluent petechiae within the infarct but without space occupying effect (HI-2). Parenchymal hematoma was defined as hematoma with slight space occupying effect in less than 30% of the infarct (PH-1) or hematoma more than 30% of the infarct with substantial space occupying effect or any hemorrhagic outside the infarcted area (PH-2) (Berger et al., 2001). Follow-up CT examination was performed 72 h after initial CT imaging in case of clinical worsening to identify HT.

The MR images were reviewed by two senior radiologists. When the two radiologists disagreed over the MR images findings, they reach a consensus after discussion. All images were exported in digital imaging and communications in medicine (DICOM) format. The largest infarction slice from MR images of each patient was chosen as the single representative slice. The regions of interest (ROIs) containing the selected slices were manually delineated on the MR images. Further image analyses were performed based on the ROIs. Texture analysis was performed using the publicly accessed software, MaZda (Institute of Electronics, Technical University of Lodz, version 4.6) (Szczypinski et al., 2009). Image texture analysis was performed on the ROIs. The best 11 texture features were extracted as a feature group for each MRI sequence based on the GLCM approach. In MaZda, the GLCM was computed for a distance of each pixel (*d* = 1) in four directions (θ = 0°, 45°, 90°, and 135°). In total, 11 second-order statistics parameters were extracted from the GLCM: angular second moment (f1), contrast (f2), correlation (f3), sum of squares (f4), inverse difference moment (f5), sum average (f6), sum variance (f7), sum entropy (f8), entropy (f9), difference variance (f10), difference entropy (f11). The mean values of the distances in four directions were recorded for each image-quality parameter.

### Assessment of the prediction model for hemorrhagic transformation based on magnetic resonance imaging-based texture features

In this study, texture features derived from DWI images were selected as potential predictors of outcomes by logistic regression analysis, and a prediction model for HT was developed. Using the caret package in R (version 3.3.2), 98 samples were randomly divided into a train cohort (*n* = 69) and test1 cohort (*n* = 29) at an approximate ratio of 7:3. Subsequently, all 98 samples were assigned to the test2 cohort (*n* = 98). In the train cohort, a multivariable logistic regression model was undertaken to construct a novel radiomics-based diagnostic model based on a set of texture features of great significance extracted from DWI (Specific parameters were as follows: “family = binomial,” direction = “backward”). Thereafter, the “predict” function in R was applied to evaluate the diagnostic abilities of our radiomics-based model in the training, test1, and test2 datasets. The “plot.roc” function in R was employed to visualize the results of receiver-operator characteristic (ROC) in the training, test1, and test2 datasets. Of note, the larger the area under the curve (AUC), the higher the diagnostic value of the model is. Calibration of radiomics signature in the prediction of HT was assessed with a calibration curve based on the “lrm” and “calibrate” functions in R. Further, decision curve analysis (DCA) was used to assess the clinical efficiency of radiomics signature in predicting HT by calculating the net benefit across a spectrum of threshold probabilities based on the “ggDCA” package in R.

### Statistical analysis

Statistical analysis was performed using Statistic Package for Social Science (SPSS) software version 23.0. Normality of the data was tested using the Shapiro-Wilk test. Normally distributed continuous variables are presented as the means and standard deviations, while non-normally distributed variables are presented using median and interquartile range (IQR) values. Categorical variables were expressed as counts and percentages. T-tests, Chi-square, Mann–Whitney U test, and Wilcoxon rank sum test were used to identify variables associated with HT. A *p*-value less than 0.05 was considered statistically significant. ROC curve analyses were performed to compare the diagnostic performance of texture features based on DWI and T2-FLAIR images and to calculate the area under the curve (AUC) for measure diagnostic accuracy for prediction HT. The higher AUC values (AUC ≥ 0.8) indicate a better prognosis prediction for HT. AUC is interpreted as follows: 0.5 < AUC ≤ 0.7 indicates low accuracy, 0.7 < AUC ≤ 0.9 indicates moderate accuracy, 0.9 < AUC < 1.0 indicates high accuracy, and AUC = 1 indicates perfect (Greiner et al., 2000).

## Results

### Characteristics of the patients

The baseline demographic data and clinical data of the two groups were summarized in Table 1. Of these 98 patients with acute MCI, 55 were males and 43 were females. Forty four patients (44.9%) developed HT with a mean age of 68.97 (9.53) years, and 54 (55.1%) patients were in the non-HT group with a mean age of 65.75 (14.35) years. There was no difference in the baseline demographic factors (age and gender), smoking, alcohol intake, hypertension, diabetes mellitus, coronary heart disease, lipid levels, and laboratory findings between the HT group and the non-HT group. The proportion of patients with atrial fibrillation among the HT group was 56.8%, significantly higher than that of patients without HT (16.7%) (*P* < 0.05). The mean baseline NIHSS was higher in the HT group than in the non-HT group (13.83 ± 4.66 vs. 9.17 ± 5.76, *p* = 0.021). With regards to intracranial arterial stenosis (ICAS), 14 (31.8%) of 44 patients who progressed to HT had symptomatic ICAS, but a higher proportion of the patients in non-HT group presented with symptomatic ICAS (61.1%, 33/54). Of the HT group, 27 (61.4%) patients were anterior circulation ischemia, and 17 (38.6%) patients were posterior circulation ischemia confirmed by MRI. In the non-HT group, 32 (59.3%) patients were anterior circulation ischemia, and 22 (40.7%) patients were posterior circulation ischemia. Of note, there was no significant difference in HT incidence between the anterior circulation and posterior circulation ischemia group. All patients were treated with single antiplatelet therapy. For the patients with hemorrhagic transformation, the antiplatelet agents (aspirin or clopidogrel) were stopped at once. All the patients received mannitol treatment to reduce cerebral edema, and appropriate rehabilitation guidance according to the clinical condition.

**TABLE 1:** Baseline demographic and clinical characteristics of patients with and without hemorrhagic transformation (HT).

Variable	HT (*n* = 44)	Non-HT (*n* = 54)	*P*-value
Age, years	68.97 ± 9.53	65.75 ± 14.35	0.238
**Sex**			
Male	24 (54.5)	31 (57.4)	0.839
Female	20 (45.5)	23(42.6)	
**Medical history**			
Hypertension	32 (72.7)	44 (81.5)	0.338
Diabetes mellitus	9 (20.5)	18 (33.3)	0.179
Atrial fibrillation	25 (56.8)	9 (16.7)	0.000
Coronary heart disease	6 (13.6)	3 (5.5)	0.292
Hyperlipemia	3 (6.8)	7 (13.0)	0.504
Hyperuricemia	2 (4.5)	8 (14.8)	0.178
Smoking	16(36.4)	16 (29.6)	0.521
Alcohol intake	3 (6.8)	8 (14.8)	0.336
**Stroke severity**			
NIHSS score on admission	13.83 ± 4.66	9.17 ± 5.76	0.021
**Findings on admission**			
Systolic blood pressure, mmHg	141.92 ± 17.35	145.28 ± 23.42	0.876
Diastolic blood pressure, mmHg	87.04 ± 11.48	86.11 ± 11.97	0.737
Total cholesterol, mmol/L	4.15 ± 1.32	4.16 ± 1.13	0.882
LDL-cholesterol, mmol/L	2.13 ± 0.77	2.15 ± 0.73	0.725
FIB, g/L	3.91 ± 1.43	3.68 ± 1.22	0.762
D-dimer, ug/mL	0.95 ± 1.21	0.98 ± 1.14	0.813
Hcy, umol/L	10.06 ± 5.78	9.94 ± 3.62	0.231
Symptomatic ICAS ≥ 50%	14 (31.8)	33 (61.1)	0.004

HT, hemorrhagic transformation; NIHSS, National Institutes of Health Stroke Scale; LDL, low-density lipoproteins; FIB, fibrinogen; Hcy, homocysteine; ICAS, intracranial arterial stenosis.

### Texture analysis to classify individuals with hemorrhagic transformation or without hemorrhagic transformation

**FIGURE 2:**
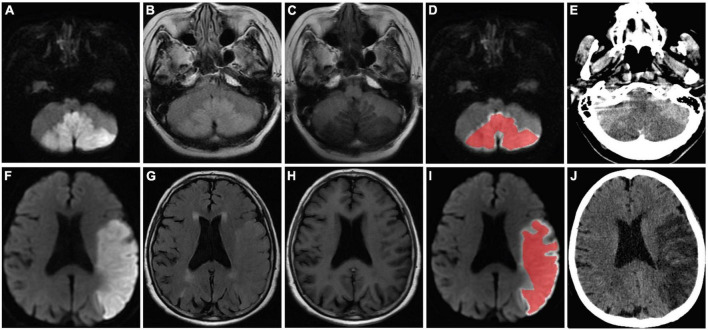
MR images of two patients (a 58-year-old female patient in no-HT group and a 75-year-old female patient who developed HT) with acute MCI. The stroke lesions of the two patients were visible as hyperintensity on DWI **(A,F)** and T2/FLAIR **(C,H)**, and hypointensity signal on T1-weighted imaging **(B,G)**, respectively. Regions of interest were placed within the infarct lesion **(D,I)** and were manually delineated to include the largest stroke lesion area (red curve). Follow-up CT was performed 72 h after the MR imaging and showed no evidence of HT **(E)**. Follow-up CT revealed infarcts with HT in the left cerebral hemisphere **(J)**. HT, hemorrhagic transformation; MCI, massive cerebral infarction; MR, magnetic resonance; DWI, diffusion weighted imaging; T2/FLAIR, T2-weighted-fluid-attenuated inversion recovery; CT, computed tomography.

**FIGURE 3:**
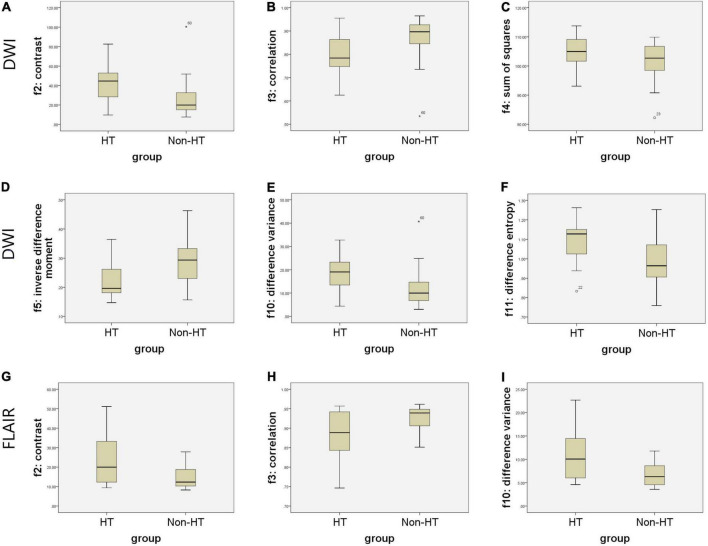
Boxplots of the most distinguishing texture parameters for predicting hemorrhagic transformation (HT) based on magnetic resonance (MR) images. Boxplots show six texture parameters (f2, f3, f4, f5, f10, and f11) with significant distinguishing capacity between infarct lesions of two groups for predicting HT based on diffusion weighted imaging (DWI) **(A–F)**. Boxplots show three texture parameters (f2, f3, and f10) with significant distinguishing capacity between infarct lesions of two groups for predicting HT based on T2-weighted-fluid-attenuated inversion recovery (T2/FLAIR) **(G–I)**.

**FIGURE 4:**
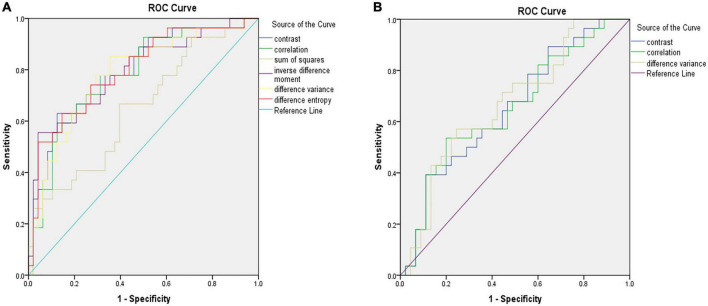
ROC analysis showing the best model for predicting hemorrhagic transformation (HT) in patients with acute massive cerebral infarction (MCI). Illustration of the accuracy of six texture analysis features (f2, f3, f4, f5, f10, and f11) in terms of ROC curves for predicting HT based on diffusion weighted imaging (DWI) **(A)**. Illustration of the accuracy of three texture analysis features (f2, f3, and f11) in terms of ROC curves for predicting HT based on T2-weighted-fluid-attenuated inversion recovery (T2/FLAIR) **(B)**. ROC, receiver operator characteristic.

Figure 2 shows the selected region of interest drawings from the brain MRI of two unrelated female patients with and without HT, respectively. The median values of 11 textural features extracted from ROIs of patients in the HT and non-HT groups are summarized in Table 2. Among the 11 textural parameters extracted from DWI images, six parameters (contrast, correlation, sum of squares, inverse difference moment, difference variance, and difference entropy) differs significantly between the two groups (*p* < 0.05) (Figure 3). Compared with non-HT group, the median contrast in DWI images was significantly increased in HT patients (44.652 vs. 19.963, *p* = 2.363 × 10^–5^), and the median correlation in DWI images was lower in HT patients (0.784 vs. 0.897, *p* = 6.450 × 10^–5^). The median difference variance and difference entropy were significantly increased in HT cohort compared with the values in non-HT patients (19.096 vs. 9.971, *p* = 6.156 × 10^–5^ and 1.128 vs. 0.964, *p* = 2.141 × 10^–5^, respectively). Our results showed overall good predictive performances of HT in f2 (contrast), f3 (correlation), f4 (sum of squares), f5 (inverse difference moment), f10 (difference variance), and f11 (difference entropy) parameters extracted from DWI images with the AUC values of 0.796, 0.779, 0.652, 0.792, 0.780, and 0.797, respectively (Figure 4). Among these features, f2 (contrast), f5 (inverse difference moment), and f11 (difference entropy) had better discriminatory power in the prediction of HT than other features (AUC > 0.7). Among these features, the AUC value of f11(difference entropy) was the highest diagnostic accuracy (AUC = 0.797, sensitivity 63%, specificity 85.4%). As shown in Table 2, based on the T2-FLAIR images, three of eleven texture features (contrast, correlation, and difference variance) were significant different between the two groups (*p* < 0.05). Compared with HT patients, the median infarct f2 (contrast) and f10 (difference variance) were significantly reduced in non-HT patients (19.997 vs. 12.319, *p* = 0.029 and 10.060 vs. 6.297, *p* = 0.015, respectively). However, the median f3 (correlation) was significantly increased in non-HT patients compared with HT patients (0.888 vs. 0.939, *p* = 0.029). The area under the curve (AUC) of combined texture features is shown in Table 2. The AUC values of f2 (contrast), f3 (correlation), and f10 (difference variance) were 0.652, 0.652, and 0.670, respectively (Figure 4). Among these features, the AUC value of f10(difference variance) was the highest diagnostic accuracy (AUC = 0.670, sensitivity 53.6%, specificity 77.8%). In present study, the texture analysis based on T2 FLAIR images were less helpful and accurate in predicting HT when compared with those upon DWI images.

**TABLE 2:** Comparison of textural parameters of diffusion-weighted image (DWI) and T2-weighted-fluid-attenuated inversion recovery (T2/FLAIR) images between hemorrhagic transformation (HT) and non-HT groups and receiver operator characteristic (ROC) analysis.

	DWI					T2WI-FLAIR				
		
	HT	Non-HT	Z-value	*P*-value	AUC	HT	Non-HT	Z-value	*P*-value	AUC
Angular second moment	0.003	0.003	−1.921	0.055	0.634	0.003	0.003	−1.929	0.054	0.635
Contrast	44.652	19.963	−4.227	0.000	0.796	19.997	12.319	−2.178	0.029	0.652
Correlation	0.784	0.897	−3.995	0.000	0.779	0.888	0.939	−2.178	0.029	0.652
Sum of squares	104.963	102.704	−2.174	0.030	0.652	105.991	103.206	−1.497	0.134	0.605
Inverse difference moment	0.197	0.294	−4.172	0.000	0.792	0.304	0.328	−1.463	0.143	0.602
Sum average	65.460	65.738	−1.203	0.229	0.584	65.109	65.092	−1.021	0.307	0.571
Sum variance	376.335	385.154	−1.148	0.251	0.580	394.778	396.678	−0.250	0.803	0.517
Sum entropy	1.833	1.864	−2.075	0.058	0.645	1.881	1.883	−0.113	0.910	0.508
Entropy	2.644	2.631	−0.806	0.420	0.556	2.708	2.655	−1.497	0.134	0.637
Difference variance	19.096	9.971	−4.007	0.000	0.780	10.060	6.297	−2.428	0.015	0.670
Difference entropy	1.128	0.964	−4.250	0.000	0.797	0.954	0.885	−1.645	0.100	0.615

DWI, diffusion-weighted image; T2/FLAIR, T2-weighted-fluid-attenuated inversion recovery; ROC, receiver operator characteristic; AUC, area under the ROC curve; HT, hemorrhagic transformation; P-value indicates differences of texture features between patients with and without HT. P < 0.05 was considered statistically significant.

### Efficacy of the magnetic resonance imaging-based prediction model in predicting hemorrhagic transformation

**FIGURE 5:**
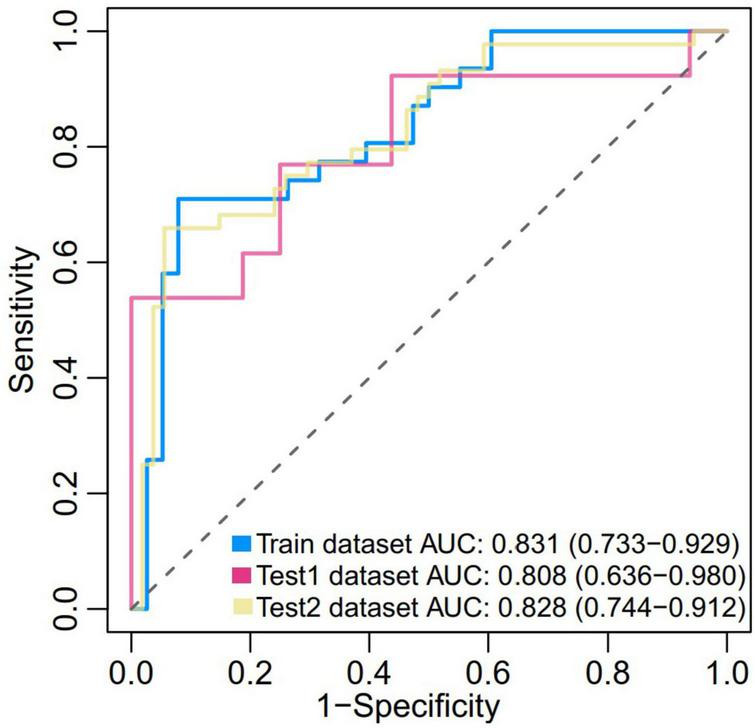
Receiver operator characteristic (ROC) of the prediction model based on a set of six different texture features (f2, f3, f4, f5, f10, and f11) of great significance extracted from diffusion weighted imaging (DWI) in both the train (0.831, [0.733, 0.929]), test1 (0.808, [0.636, 0.980]), and test2 dataset (0.828, [0.744, 0.912]).

**FIGURE 6:**
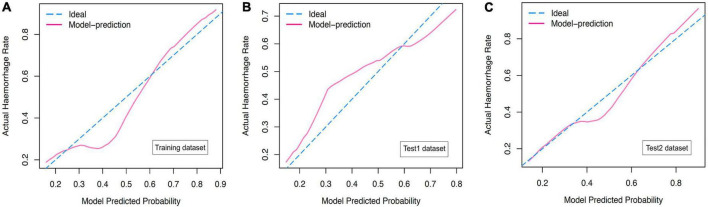
**(A)** The calibration curves for diffusion weighted imaging (DWI)-based prediction model in the training dataset. **(B)** The calibration curves for DWI-based prediction model in the test1 dataset. **(C)** The calibration curves for DWI-based prediction model in the test2 dataset.

**FIGURE 7:**
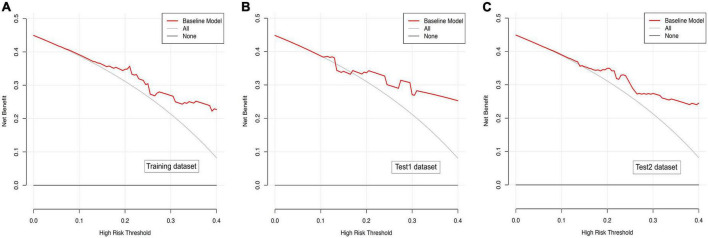
**(A)** Decision curve analysis for diffusion weighted imaging (DWI)-based prediction model in the training dataset. **(B)** Decision curve analysis for DWI-based prediction model in the test1 dataset. **(C)** Decision curve analysis for DWI-based prediction model in the test2 dataset.

In the present study, we constructed a novel radiomics panel based on a set of 6 different texture features (contrast, correlation, sum of squares, inverse difference moment, difference variance, and difference entropy) of great significance extracted from DWI for predicting HT in patients with acute MCI, and analyze the discrimination and calibration of this model. For the train, test1, and test2 cohorts, the AUC values of ROC curves (Figure 5) produces satisfactory results, indicating our radiomics-based panel could serve as an excellent diagnostic indicator of HT. Likewise, the calibration curves (Figure 6) and DCA plots (Figure 7) validated the clinical usefulness of our radiomics-based panel in differentiating patients with HT from non-HT patients.

## Discussion

In this study, we investigated the performance of texture analysis based on MR images in predicting HT in patients with acute MCI. To the best of knowledge, this is the first study in which the predictive ability of MRI-based texture analysis is evaluated in patients with acute MCI. Our results show that texture analysis has a potential power to distinguish infarcted tissues that are prone to develop HT from the surrounding tissues.

The pathophysiology mechanisms of HT after ischemic stroke is still unclear. Recent studies show evidences of significant hemodynamic and metabolic imbalances led to breakdown of blood-brain barrier and eventually increased extravasation of blood (Warach and Latour, 2004; Hong et al., 2021). Accurate prediction of HT after AIS is crucial for therapeutic regimen selections on patient’s prognosis to reduce the occurrence of HT. Many risk factors or predictors have been described for HT after AIS. Although several predictors of HT have been identified in AIS, the association between these predictors and HT remains controversial. Reperfusion therapy, including intravenous thrombolysis with rt-PA or mechanical thrombectomy, are well-established risk factors for HT in patients with AIS. To avoid the influence of reperfusion therapy on HA, only those acute MCI patients who did not receive intravenous rt-PA as well as mechanical thrombectomy were included in this study. Hypertension, diabetes mellitus, and hematological parameters did not contribute to HT in our study, consistent with previous studies (Bang et al., 2007; Yu et al., 2018). The mean baseline NIHSS for participants in the HT group was significantly higher than that of patients without HT. In agreement with a previous study, the distribution of atrial fibrillation was significantly higher in patients with HT than in patients without HT (Paciaroni et al., 2008). There is growing evidence showing that atrial fibrillation is related to a lower recanalization rate after thrombolysis but increased risk for HT (Dang et al., 2019). In our study, the relatively lower frequency of ICAS in HT group compared with non-HT group, which indicates that the patients with symptomatic ICAS were less likely to develop HT. This may be due to prolonged or impaired downstream perfusion as a result of inadequate collateral compensation (Feng et al., 2019).

Moreover, advanced neuroimaging techniques have recently been wide applied to study ischemic stroke because of its capacity to predict HT. Quantitative texture analysis based on the MR sequences, such as DWI, T2/FLAIR or PWI, has been used for the prediction of HT in AIS. However, the feasibility and effectiveness of MRI-based texture analysis in order to evaluate HT in patients after ischemic stroke remains largely untested. Texture analysis is a well-established radiology techniques that detects the invisible signal changes among image pixels. It may reduce the degree of variability in imaging interpretation that relies primarily on human visual perception (Kassner et al., 2009). MRI-based texture analysis can be used to quantify the spectral properties, pixel interrelationships, gray-level patterns of MRI images, and the spatial distribution differences of lesions within the brain parenchyma. MRI-based texture analysis is able to reveal ischemic lesions and can be used to assess the severity of ischemic stroke (Huisa et al., 2013; Wang et al., 2020). Kassner et al. (2009) demonstrated that texture analysis based on postcontrast T1-weighted images is a good predictor of HT in AIS. However, the prediction of HT by MRI-based texture analysis in acute MCI has not been reported so far. Our results showed overall good predictive performances of HT in textural parameters extracted from DWI images with the higher values of AUC. Although three textural parameters on T2/FLAIR images reached statistical significance, we failed to find any features with high diagnostic power between images from patients with HT and patients without HT. This suggests that DWI-based texture analysis tend to have higher prediction value for HT in patients with acute MCI than the analysis base on T2/FLAIR images. Multiparametric MRI texture analysis model should be developed to improve the prediction performance of HT following AIS.

Hemorrhagic transformation is an important factor related to stroke outcome, but its influence on functional outcome is currently unclear. A prospective multicenter study in Italy reported that the risk of 24-h neurological deterioration and 3-month death was severely increased after PH (Paciaroni et al., 2008). Van Kranendonk et al. (2019) found that not only PH2 was related to functional outcome after stroke but other smaller types of HT might influence functional outcome. Moreover, the management of HT in adults with acute MCI is often more complicated and challenging. There have been no appropriate guidelines about the use of antithrombotics after HT. Accordingly, the guidelines organized by the Chinese Stroke Association recommend the temporary discontinuation of antiplatelet or anticoagulant medications in stroke patients with HT (Liu et al., 2020). Treatments of HT also include blood pressure management, reversing coagulopathy, and management of intracranial pressure. Therefore, early identification of HT in patients with acute MCI is particular important, and may be used to guide the selection of patients for thrombolytic therapy, and subsequent monitoring of HT.

In summary, our preliminary results showed that MRI-based texture has a good predictive validity for HT in patients with acute massive cerebral infarction. There are a few limitations that should be noted in the present study. First, this retrospective cohort study may have selection bias. Second, TA was performed on the largest slice rather than the whole infarcted lesion, which may have an immeasurable effect on the final results. Third, the sample size in our study was relatively small. Further investigations with a large sample size are warranted. Finally, we fail to perform MRI-based texture analysis after classification of HT because of limited number of patients with parenchymal hematoma. In spite of these limitations, MRI-based TA can facilitate the accurate prediction of HA in patients with acute MCI and can optimize the development of clinical decisions.

## Data availability statement

The original contributions presented in this study are included in the article/supplementary material, further inquiries can be directed to the corresponding authors.

## Ethics statement

The studies involving human participants were reviewed and approved by Ethics Committee of Union Hospital, Tongji Medical College of Huazhong University of Science and Technology. The patients/participants provided their written informed consent to participate in this study.

## Author contributions

HZ and ZL: methodology, data curation, software, image processing, statistical analysis, writing—original draft, and visualization. SW and ZC: software, data curation, image processing, and statistical analysis. YL and YX: conceptualization, methodology, writing—review and editing, and supervision. All authors contributed to the article and approved the submitted version.

## Conflict of interest

The authors declare that the research was conducted in the absence of any commercial or financial relationships that could be construed as a potential conflict of interest.

## Publisher’s note

All claims expressed in this article are solely those of the authors and do not necessarily represent those of their affiliated organizations, or those of the publisher, the editors and the reviewers. Any product that may be evaluated in this article, or claim that may be made by its manufacturer, is not guaranteed or endorsed by the publisher.
